# HLA-Clus: HLA class I clustering based on 3D structure

**DOI:** 10.1186/s12859-023-05297-x

**Published:** 2023-05-09

**Authors:** Yue Shen, Jerry M. Parks, Jeremy C. Smith

**Affiliations:** 1 Graduate School of Genome Science and Technology, University of Tennessee, Knoxville, TN 37996 USA; 2 Biosciences Division, Oak Ridge National Laboratory, Oak Ridge, TN 37831 USA; 3 Department of Biochemistry and Cellular and Molecular Biology, University of Tennessee, Knoxville, TN 37996 USA

**Keywords:** Human leukocyte antigen, Protein structure, Clustering, Machine learning

## Abstract

**Background:**

In a previous paper, we classified populated HLA class I alleles into supertypes and subtypes based on the similarity of 3D landscape of peptide binding grooves, using newly defined structure distance metric and hierarchical clustering approach. Compared to other approaches, our method achieves higher correlation with peptide binding specificity, intra-cluster similarity (cohesion), and robustness. Here we introduce HLA-Clus, a Python package for clustering HLA Class I alleles using the method we developed recently and describe additional features including a new nearest neighbor clustering method that facilitates clustering based on user-defined criteria.

**Results:**

The HLA-Clus pipeline includes three stages: First, HLA Class I structural models are coarse grained and transformed into clouds of labeled points. Second, similarities between alleles are determined using a newly defined structure distance metric that accounts for spatial and physicochemical similarities. Finally, alleles are clustered via hierarchical or nearest-neighbor approaches. We also interfaced HLA-Clus with the peptide:HLA affinity predictor MHCnuggets. By using the nearest neighbor clustering method to select optimal allele-specific deep learning models in MHCnuggets, the average accuracy of peptide binding prediction of rare alleles was improved.

**Conclusions:**

The HLA-Clus package offers a solution for characterizing the peptide binding specificities of a large number of HLA alleles. This method can be applied in HLA functional studies, such as the development of peptide affinity predictors, disease association studies, and HLA matching for grafting. HLA-Clus is freely available at our GitHub repository (https://github.com/yshen25/HLA-Clus).

**Supplementary Information:**

The online version contains supplementary material available at 10.1186/s12859-023-05297-x.

## Background

Human leukocyte antigen (HLA) class I proteins, which include HLA-A, HLA-B, and HLA-C, play an essential role in the adaptive immune system by presenting intrinsic peptide antigens to CD8^+^ T cells and eliciting a cytotoxic immune response [1, 2]. The extreme functional polymorphism of HLA alleles complicates the investigation of these important macromolecules [3] (Additional file 1: Fig. S1). To help disentangle this complex system of proteins, supertypes have been defined to include alleles that have similar peptide binding specificities [4, 5]. Comprehensively determining peptide binding specificities using experimental methods requires exorbitant time and effort [6–8]. Thus, in silico methods have been developed as viable alternatives including affinity prediction-based [9, 10], sequence-based [11, 12], and structure-based methods [13, 14]. Because function is determined by structure, the structure-based methods may provide advantages over sequence-based approaches. However, the accuracy and coverage of previous structure-based approaches has been limited by the lack of availability of high-quality structures, the performance of algorithms, and computational demand for the required analysis.

We recently presented an HLA class I structure-based supertype and subtype classification method that combines multiple targeted solutions [15]. We briefly summarize the approach here. By using ColabFold [16], a notebook-based implementation of AlphaFold2 [17], high-quality HLA class I structures were generated. In addition, coarse graining of protein models was applied to reduce the computational cost of structural analyses and the impact of possibly inaccurate side chain positioning introduced by modeling. Inspired by the successful application of structural similarity in comparing small molecule binding pockets [18–21], a structure distance metric, SD*,* was adapted from the atom-level point cloud-based *sup-CK* algorithm [21], which performs well in classification accuracy applied to binding pocket prediction. To incorporate physicochemical properties and the varied importance of specific HLA binding-site residues, we implemented a similarity matrix and weight factor, which improve the correlation between structural and functional similarity. Compared to previous clustering methods, our approach offers improved correlation with peptide binding specificity, intra-cluster similarity (cohesion), and cluster stability against random sampling (robustness).

Here we present a package, HLA-Clus, for clustering HLA class I alleles based on the method described above. HLA-Clus includes three major stages: structure processing, structure distance calculation, and clustering. First, the 3D HLA structures are processed into labeled point clouds. Then, the structural distances (SD) between alleles are calculated. Lastly, based on the pairwise SD matrix, alleles are clustered hierarchically using a complete linkage method. A nearest-neighbor clustering method is also available to allow new alleles to be easily incorporated into existing, predefined clusters.

As an example application, we interfaced HLA-Clus in the peptide:HLA affinity predictor, MHCnuggets, which employs allele-specific deep learning models. The affinity prediction of alleles lacking a corresponding deep learning model is achieved by selecting the closest prediction model, which is referred to as model selection, based on sequence-based allele similarity and model quality. Instead, we used HLA-Clus nearest neighbor clustering method in model selection. Compared to the default method, HLA-Clus improved the binding prediction accuracy and correlation with experimental affinity on tested alleles.

The pipeline is fully open source to enable easy use and modification by users. Recommended parameters are provided for rapid start-up. Although not demonstrated explicitly, this approach has the potential to be adapted to clustering of other classes of proteins based on structure distance metrics.

## Implementation

### Overview of structure-based HLA class I similarity measurement

The details of implemented methods have been described in detail previously [15] but are briefly summarized here.

Similarity between alleles is measured by a newly defined structure distance metric, SD, which was adapted from the Sup-CK method [21], a point cloud-based all-atom structure distance metric designed for comparing small molecule binding pockets. However, the high computational demand is a major issue when applying this method in HLA molecules. Furthermore, the Sup-CK requires a gradient ascent optimization to find the optimal relative orientation, which greatly increased the computational demand.

We modified the metric for use with coarse-grained models to improve calculation speed, incorporate weight factors representing the importance of each residue, and implement physicochemical similarity at the residue level. By coarse graining, the number of points in a structure is greatly decreased. Also, structure alignment was used to find the optimal relative orientation between HLAs, so that the gradient ascent optimization could be omitted. This is applicable because all HLA class I molecules are homologous with highly similar structures. In addition, Sup-CK uses a hard cutoff to determine the residues that contact the peptide, instead our method uses the weight factors as a soft threshold, which avoids the inaccuracy brought by falsely detected binding pocket.

The SD metric incorporates three components: spatial similarity, physicochemical similarity, and weight factor. First, the similarity K between two HLA proteins P1 and P2 is defined as:

1 $$K\left( {P1,\,P2} \right) = \mathop \sum \limits_{i \in P1} \mathop \sum \limits_{j \in P2} \sqrt {w_{i} w_{j} } \cdot S_{ij} \cdot \frac{1}{{{\text{cosh}}^{k} \left( {\sigma \cdot \left\| {x_{i} - x_{j} } \right\|} \right)}}$$

The spatial similarity is quantified by a kernel function that transforms the Euclidean distance between two pairs of residues x_i_ and x_j_ into a value between 0 and 1. The physicochemical similarity S_ij_ between two residues is measured using a transformed Grantham distance [22] matrix, which measures the physicochemical similarity between residues. The weight factors w_i_ and w_j_ were adapted from a previous study [23] that indicated the relative importance of the position in determining peptide binding affinity.

To normalize the positive definite similarity measurement K, the structure distance metric (SD) was defined as:

2 $$SD\left( {P1,\,P2} \right) = \sqrt {K\left( {P1,\,P1} \right) + K\left( {P2,\,P2} \right) - 2K\left( {P1,\,P2} \right)}$$

#### Algorithm overview

**Fig. 1:**
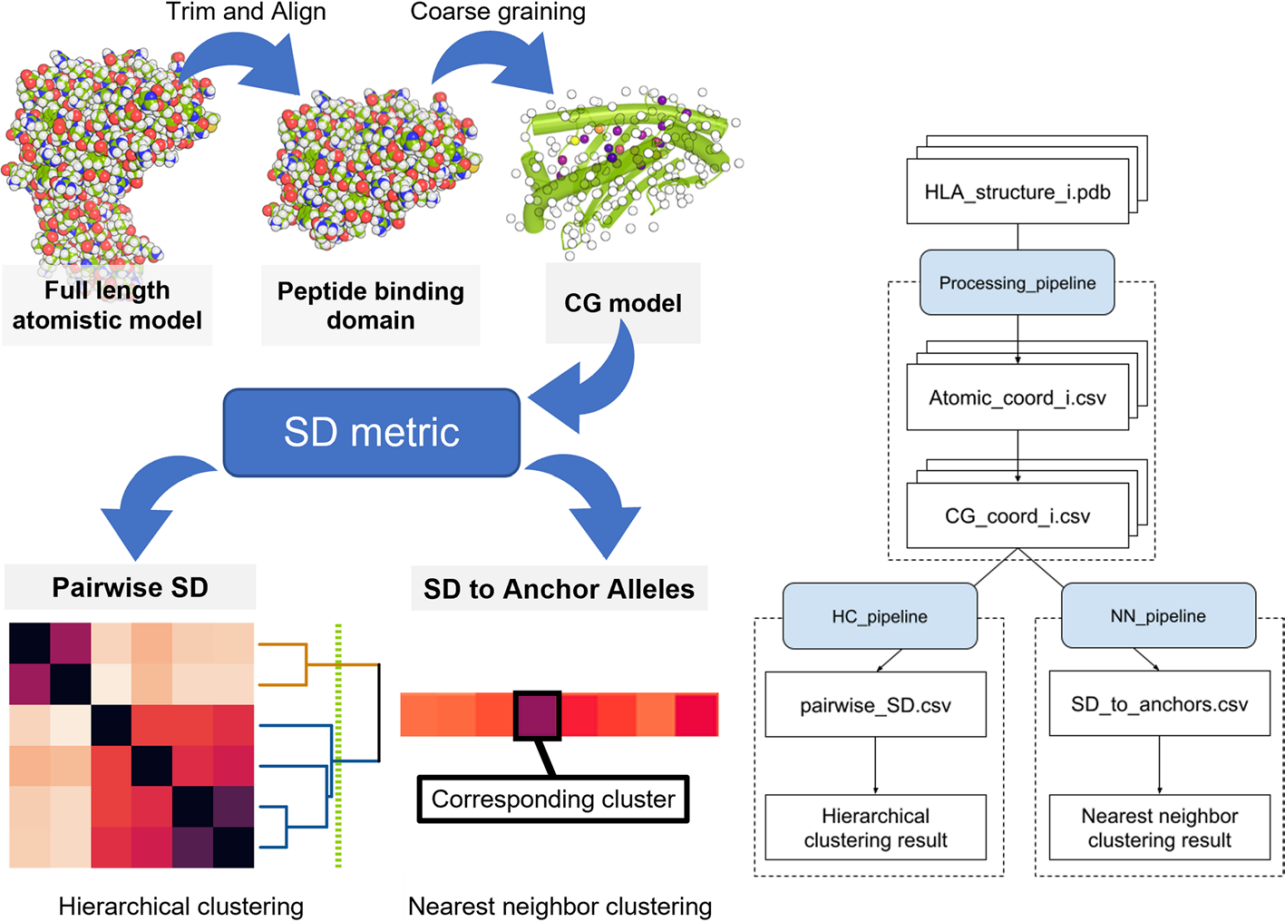
Flowchart of HLA-Clus clustering pipeline. **a** Figurative demonstration of input and output for each stage in the pipeline. **b** Schematic flowchart. The rectangles represent input and output files, while the blue parallelograms represent callable functions from HLA-Clus package. The stacked rectangle and numeral indicator (i) in file name illustrate that there are multiple files

We implemented the above-mentioned method in Python 3.8 with publicly available packages, including NumPy, SciPy, pandas, BioPandas, pymol-bundle, Biopython, matplotlib, seaborn, and scikit-learn. The source code is available in our GitHub repository [24]. Users can install the package via pip or by cloning the repository. The method has three stages: structure processing, structure distance calculation, and clustering (Fig. 1). Example outputs are available as supplemental materials (Additional file 2: Tables S1–S4).

#### Stage 1: HLA class I structure processing

The HLA-Clus package accepts 3D structures of HLA class I α chains as input. In the previous study we modeled 449 populated HLA class I alleles [15]. The structures are available at [25] for download and further investigation.

##### Trim and align

The 3D structures are trimmed to include only the 179 residues that form the peptide binding domain (residues 2-180). The trimmed models are superimposed onto the structure of the peptide binding domain of the most studied allele HLA-A*02:01 (PDB ID: 1i4f).

##### Coarse graining

The structures are coarse grained, with each residue represented by the center of mass of its side chain and the backbone atoms omitted. The coordinates and residue types are stored in a CSV file.

##### Assigning weight factors

Weight factors, which were adapted from a previous study [23], are assigned to residues by the position according to their relative importance in determining peptide binding specificity. The weight factors are recorded in the CSV files of coarse-grained structures and can be changed by passing a Python dictionary with the residue position as the key and the weight factor as the value.

##### Processed HLA structure file

Processed HLA structures are stored in CSV files. Each file includes 179 rows and 7 columns. Each row corresponds to one residue, and the columns are chain identifier, residue number, residue name, Cartesian coordinates, and weight factor.

#### Step 2: measuring similarity between alleles using structure distance (SD) metric

This step is the most time consuming in the HLA clustering pipeline, especially for the hierarchical clustering method, and so calculation speed has been optimized as follows. As the definition of SD suggests, the calculation of SD is split into two stages: the similarity score K and the structure distance SD. The similarity score K between a pair of alleles is split into three parts and calculated separately: spatial similarity, physicochemical similarity, and average weight factor. Then, the final result is generated with vectorized calculations.

##### Spatial similarity

The spatial similarity is calculated according to the kernel function. The input is a 3D coordinates with shape (179, 3), and the result is a 2D matrix with shape (179, 179).

##### Physicochemical similarity

The physicochemical similarity is derived by looking up values in the similarity matrix. The input is a (179, 1) NumPy array, and the result is a 2D matrix with shape (179, 179).

##### Average weight factor

The average weight factor is the geometric mean of the weight factors of two compared residues and is calculated as the square root of the outer product of two weight factor NumPy arrays with shape (179, 1). The result is a 2D matrix with shape (179, 179).

##### Calculation of structural similarity K

K is calculated as the grand sum of the element-wise product of the three matrices: spatial similarity, physicochemical similarity, and average weight factor.

##### Calculation of structure distance SD

As shown in Eq. 2, the calculation of SD(P1, P2) requires three components: K(P1, P1), K(P2, P2), and K(P1, P2). Because the self-similarity values (e.g., K(P1, P1)) are used multiple times and K(P1, P2) = K(P2, P1), the calculation process was optimized for efficiency.

In the hierarchical clustering mode, the SD matrix is calculated as follows:

(i) The combination with repetition of query HLA alleles is generated, so that only one of K(P1, P2) and K(P2, P1) will be calculated.
(ii) The structural similarity K for each allele pair in the combination is calculated, and the values are stored in a Python dictionary.
(iii) Finally, the elements in the SD matrix are calculated by looking up K values in the dictionary. Because the SD matrix is symmetric about the diagonal, and the diagonal is always 0, only the upper triangular portion of the SD matrix is calculated.

In the nearest neighbor clustering mode, because the query and anchor alleles differ, the output SD matrix is not symmetric, and the similarity between two anchor alleles or two query alleles is not needed. Therefore the calculation of K is divided into two cases: the self-similarity (e.g., K(P1, P1)) and anchor-query similarity (e.g., K(P1, P2)). The anchor-query similarity is calculated according to the combination of anchor-query pairs without replacement. Both similarity values are stored in a Python dictionary. Finally, the SD matrix is calculated by looking up K values.

Besides the optimization of the calculation process, a multiprocessing method was implemented to improve calculation speed. In the calculation of structural similarities, the K value of each pair of alleles is calculated in parallel.

#### Step 3: clustering of alleles based on SD

Two clustering methods are available. The hierarchical clustering method clusters all query alleles, while the nearest-neighbor clustering approach is used to cluster query alleles according to an existing or user-defined clustering scheme.

##### Hierarchical clustering

The hierarchical clustering method first calculates the pairwise SD matrix between alleles to be clustered. Then, hierarchical clustering is performed with a user-defined number of clusters and linkage method.

##### Nearest neighbor clustering

To use the nearest-neighbor approach, anchor alleles and corresponding clusters must be defined. An anchor allele is the structure used as a representative of a predefined cluster (Table 1). For each query allele awaiting clustering, the SD to each anchor allele is calculated, and the query allele is assigned to the cluster represented by the nearest anchor allele.

**Table 1:** Default anchor alleles and corresponding supertypes/subtypes for nearest-neighbor clustering. The classification is derived from our previous study [15]

Anchor allele	Subtype	Supertype
HLA-A*01:01	A01	A01-A03-A66
HLA-A*03:01	A03	
HLA-A*11:01		
HLA-A*30:01		
HLA-A*66:01	A66	
HLA-A*02:01	A02	A02
HLA-A*02:03		
HLA-A*02:06		
HLA-A*02:07		
HLA-A*68:01		
HLA-A*24:02	A24	A24
HLA-B*07:02	B07	B07-B35
HLA-B*42:01		
HLA-B*35:01	B35	
HLA-B*08:01	B08	B08-B18-B39
HLA-B*18:01	B18	
HLA-B*39:01	B39	
HLA-B*14:02	B14	B14
HLA-B*15:01	B15	B15-B40
HLA-B*40:02		
HLA-B*40:01	B40	
HLA-B*27:05	B27	B27
HLA-B*44:02	B44	B44
HLA-B*44:03		
HLA-B*51:01	B51	B51-B58
HLA-B*57:01	B58	
HLA-B*58:01		
HLA-C*04:01	C01	C01-C02
HLA-C*05:01		
HLA-C*08:02		
HLA-B*46:01	C02	
HLA-C*06:02		
HLA-C*07:01	C07	C07

#### Choice of optimal number of clusters (N) for hierarchical clustering

To select the optimal number of clusters, the elbow method and silhouette method have been implemented. First, hierarchical clustering is performed given multiple consecutive numbers of clusters (N) and then the sum of squared errors (SSE) and the silhouette coefficient (SC) are calculated for each clustering result.

The SSE is defined as the sum of distances between alleles and corresponding cluster centers, which is conventionally the average of cluster members. However, for a precomputed pairwise distance matrix, this average is not preferred because it may be unphysical. Therefore, we instead use cluster centroids, which are calculated as:

$$SSE = \mathop \sum \limits_{C} \mathop \sum \limits_{i \in C} SD\left( {i,\,centroid\left( C \right)} \right)$$

The silhouette coefficients are calculated in several steps. First, for each allele *i* that belongs to cluster *C*, the average distance to all other alleles in the same cluster is defined as:

$$a\left( i \right) = \left\{ {\begin{array}{*{20}l} {\frac{1}{size\left( C \right) - 1}} \hfill & {\mathop \sum \limits_{i,j \in C, i \ne j} distance\left( {i,j} \right), \,size\left( C \right) > 1} \hfill \\ {0,} \hfill & {size\left( C \right) = 1} \hfill \\ \end{array} } \right.$$

Next, the average distance to the closest neighboring cluster *D* for each allele *i* is defined as:

$$b\left( i \right) = \mathop {\min }\limits_{C \ne D} \frac{1}{size\left( D \right)}\mathop \sum \limits_{k \in D} distance\left( {i,k} \right)$$

Lastly, the SC for the clustering result with *n* samples is calculated using the following equation:

$$SC = \frac{1}{n}\mathop \sum \limits_{i}^{N} \frac{b\left( i \right) - a\left( a \right)}{{\max \left( {a\left( i \right),b\left( i \right)} \right)}}$$

The elbow plot (SSE vs N) and silhouette plot (SC vs N) are generated using the Matplotlib library. The optimal value of N can be selected from elbow points (i.e., the inflection point) of the SSE curve or the peaks (i.e., local maxima) of the silhouette curve.

#### Test example: model selection for MHCnuggets using HLA-Clus nearest-neighbor clustering

To demonstrate the application of HLA-Clus in characterizing similarities between HLA alleles, we implemented our nearest-neighbor clustering approach in the MHCnuggets pipeline. The peptide:MHC affinity predictor MHCnuggets [26] includes allele-specific deep learning models for 102 classical HLA class I alleles. However, affinity prediction for rare alleles lacking a corresponding deep learning model is performed by using the model of the closest well-characterized allele. This procedure is referred to as model selection. Thus, the predictive performance of MHCnuggets on rare alleles is determined by the quality of both the deep learning-based affinity prediction model and the model selection algorithm. We implemented HLA-Clus to replace the default model selection in MHCnuggets and compared its performance to the default algorithm.

The default algorithm was assessed previously by the authors using a leave-one-molecule-out (LOMO) test. In the original LOMO test protocol, 20 well characterized alleles were chosen as pseudo-rare alleles (“LOMO allele”). Then, for each of the 20 alleles the data in the training set for that allele was held out, and deep learning models were trained using data from all other alleles. Finally, the affinity of the held-out peptides was predicted by the remaining models and compared to the held-out experimental data.

Because MHCnuggets has been updated over the years, we reimplemented the LOMO test that was used in the default model selection method. We used the original dataset for the LOMO test containing experimental affinities for peptides binding to 20 alleles, referred to as *IEDB class I rare alleles*. Instead of retraining the deep learning models for each of the LOMO alleles, the model was selected using the *closest_allele* function in the *find_closest_mhcI.py* script by omitting the tested allele one allele at a time in the model search file *examples_per_allele.pkl* from the MHCnuggets source code.

For comparison, we applied the HLA-Clus nearest-neighbor clustering method to select the closest model. Among the 102 alleles that have a prediction model, four are invalid according to the IPD-IMGT/HLA database (version 3.50), including three LOMO alleles, and were therefore excluded from further analysis. Structural models of the 98 valid alleles were generated using ColabFold as described previously [15]. Next, each of the remaining 17 alleles was clustered using the nearest-neighbor method together with the remaining 97 alleles, and the closest model was selected according to the clustering result.

Finally, the affinity of peptides to the 17 alleles was predicted using the closest model trained on binding affinity data (i.e., *ba_models* = True) and compared to the corresponding experimental result. The performance was assessed by binding prediction accuracy and correlation between the predicted and experimental IC_50_ values. The binding prediction, binder:nonbinder binary classification was based on an IC_50_ threshold of 500 nM. The accuracy was calculated as the number of peptides that have identical binding results (i.e., binder or non-binder) between predicted and experimental values divided by the total number of tested peptides. The correlation between predicted and experimental values was calculated as the Spearman rank correlation coefficient.

## Results

### Test example: application of HLA-Clus to MHCnuggets model selection improves peptide binding prediction accuracy for rare alleles

As an example of the use of HLA-Clus in identifying similar alleles for use in peptide binding affinity classification, we demonstrate the application of a newly added nearest-neighbor clustering method in the MHCnuggets package and test its performance. HLA-Clus provides an SD metric for predicting the similarity of peptide binding specificity between HLA class I alleles and two clustering methods: hierarchical clustering and nearest-neighbor clustering. The performance of the SD metric and hierarchical clustering method was demonstrated in our previous article [15].

The MHCnuggets package predicts peptide:HLA affinity using allele-specific deep learning models. To make predictions for rare alleles, the closest deep learning model is used, which is selected by a sequence-based algorithm. As we demonstrated previously [15], our structure-based method has a higher correlation with peptide binding specificity than does a sequence-based comparison. We now investigate if HLA-Clus improves model selection relative to the default method.

**Fig. 2:**
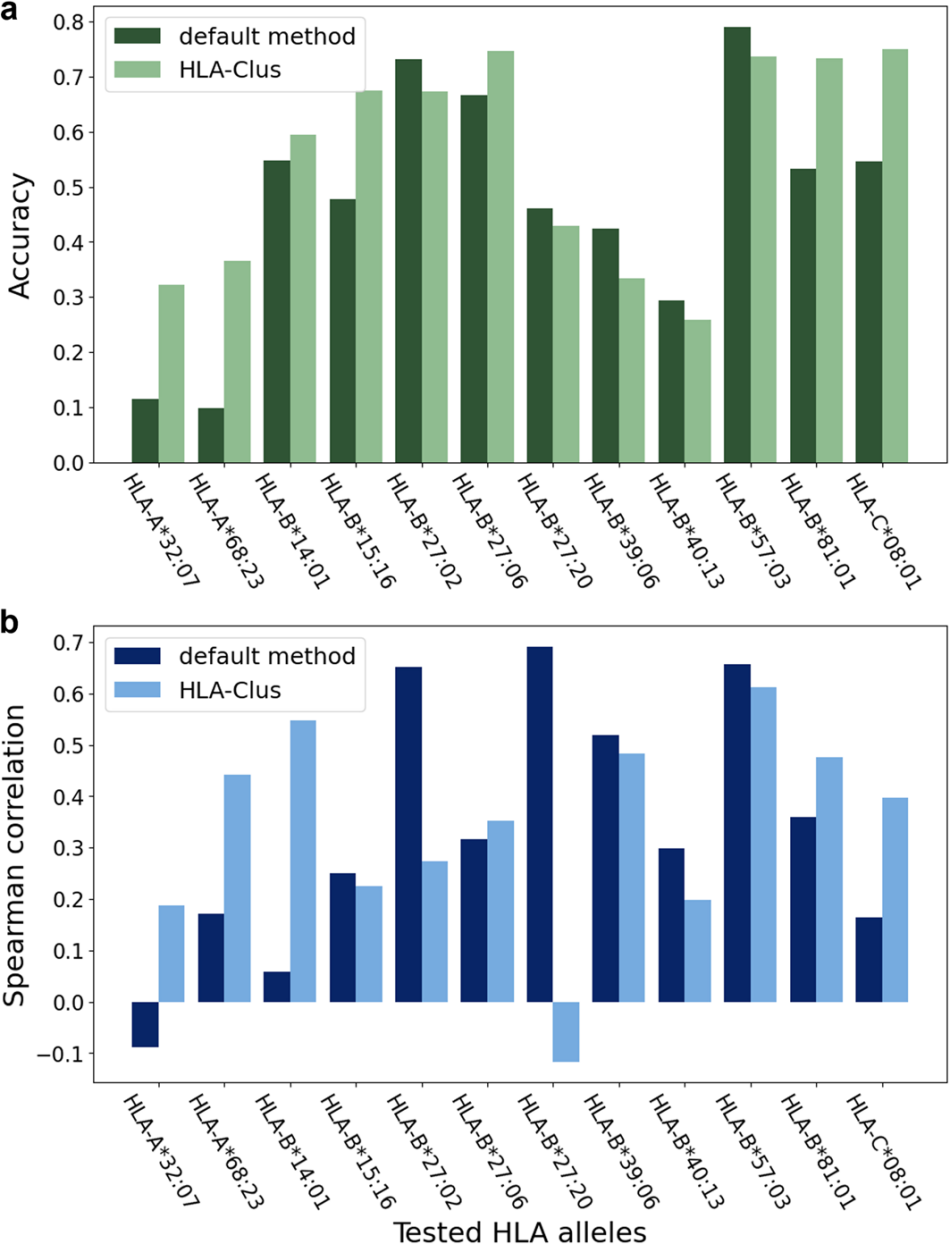
Performance of the default method in ref 24 and HLA-Clus model selection methods in the LOMO test assessed by (**a**) binder:non-binder classification accuracy and (**b**) predicted:experimental affinity correlation

In the model selection result, among the 17 valid LOMO alleles, 12 alleles were assigned different closest models by the two methods. We further compared the performance of MHCnuggets on these 12 alleles using two groups of closest models given by HLA-Clus and the default method, via accuracy of binder:non-binder binary classification and the Spearman rank correlation coefficient. On average, the HLA-Clus group shows higher accuracy in binding prediction than the group obtained with the default method (Fig. 2a), as the average accuracy of HLA-Clus group is 0.55 compared to 0.47 using the default method (Table 2). Among the 12 alleles, seven have improved accuracy while five show a decrease. The Spearman correlation coefficient shows no significant difference on average (Table 2), while for each individual allele, the correlation coefficient varies significantly (Fig. 2b). In general, the classification accuracy and correlation coefficient are positively correlated, as a good predictor is expected to perform well on both correlation and classification scenarios.

**Table 2:** Binary classification performance from Leave-One-Molecule-Out Tests for 12 HLA Class I alleles that were assigned different closest models by the default model selection algorithm in MHCnuggets and HLA-Clus

LOMO test HLA allele^a^	Default				HLA-Clus			
	Closest model^b^	Training set size	Accuracy	Correlation	Closest model	Training set size	Accuracy	Correlation
A*32:07	A*01:01	6209	0.11	− 0.09	A*31:01	6280	0.32	0.19
A*68:23	A*03:01	9651	0.10	0.17	A*68:01	4385	0.37	0.44
B*14:01	B*27:05	4402	0.55	0.06	B*14:02	223	0.60	0.55
B*15:16	B*58:01	4422	0.48	0.25	B*15:17	1452	0.67	0.23
B*27:02	B*27:05	4402	0.77	0.65	B*27:03	912	0.67	0.27
B*27:06	B*27:05	4402	0.69	0.32	B*27:20	91	0.75	0.35
B*27:20	B*27:05	4402	0.46	0.69	B*27:06	87	0.43	-0.12
B*39:06	B*27:05	4402	0.42	0.52	B*39:01	1692	0.33	0.48
B*40:13	B*40:01	4337	*0.29*	0.30	B*40:02	1087	0.26	0.20
B*57:03	B*58:01	4422	*0.79*	0.66	B*57:01	2914	0.74	0.61
B*81:01	B*07:02	6938	*0.53*	0.36	B*42:01	218	0.73	0.48
C*08:01	C*08:02	135	*0.55*	0.16	C*03:03	164	0.75	0.40
Average			0.47	0.34			0.55	0.34

^a^For simplicity, the “HLA-” prefix was omitted for all alleles in this table

^b^The closest allele was not reported in ref 24 but was identified here by applying the procedure described therein

We further investigated the potential cause of the cases in which the prediction performance decreased when HLA-Clus was used by examining the number of peptides in the training data for each model contained in the *examples_per_allele.pkl* file in the MHCnuggets source code. In most cases (10 out of 12), the models selected by HLA-Clus have a much smaller training set than the default algorithm, especially for the five alleles that show a decrease in prediction performance (Table 2). In the extreme example, the tested allele HLA-B*27:20, the default method selected model HLA-B*27:05 includes 4402 peptides in the training set, while HLA-Clus selected HLA-B*27:06, which contains only 87 peptides in the training set. Therefore, we conclude that the insufficient training data is the main cause of the decreased performance using HLA-Clus to identify the closest allele. On the other hand, this finding also suggests the advantage of HLA-Clus over the default method, as a better prediction performance was achieved using much smaller training sets. By combining HLA-Clus with the consideration of model quality applied in the default MHCnuggets model selection algorithm, a substantial improvement in performance is expected.

## Conclusions

Here we presented the HLA-Clus package for clustering HLA class I alleles with similar peptide binding specificities based on similarity of the peptide binding groove landscape. The clustering pipeline first processes modeled 3D HLA structures into coarse-grained point clouds. It then calculates the pairwise SD matrix between HLA alleles and clusters alleles into groups using a hierarchical or nearest-neighbor method. The structure distance metric SD correlates strongly with the peptide binding specificity, leading to reliable supertype and subtype classification [15].

In addition, HLA-Clus is versatile and can be readily applied in various scenarios. For example, we have demonstrated that using the nearest-neighbor clustering method in HLA-Clus can improve peptide binding prediction in MHCnuggets for rare alleles by upgrading the model selection algorithm. Moreover, HLA-Clus has the potential to be used in disease association studies to merge similar alleles into groups for streamlining analyses. It may also be useful for HLA matching in transplantation studies (Additional file 1: Fig. S1 and Additional file 2: Tables S1–S4).

## Availability and requirements

Project name: HLA-Clus.

Project home page: https://github.com/yshen25/HLA-Clus.

Operating system(s): Platform independent.

Programming language: Python.

Other requirements:

License: GPL-3.0

## Supplementary Information

**Additional file1**. **Figure S1**: Comparison between the number of HLA class I alleles studied previously.

**Additional file2**. **Table S1**: Example output of the Processing_pipeline function. **Table S2**: Example output of HC_pipeline function. **Table S3**: Example of anchor_dictionary parameter for NN_pipeline function. **Table S4**: Example output of NN_pipeline output.

## Data Availability

The HLA-Clus package is available at https://github.com/yshen25/HLA-Clus. The functions and scripts were written in Python 3 using publicly available packages. The clustering pipeline and examples are also provided as Jupyter notebooks. The datasets supporting the conclusions of this article are included within the article and its additional files.

## Abbreviations

HLA: Human leukocyte antigen
3D: Three-dimensional
SD: Structure distance
SSE: Sum of squared errors
SC: Silhouette coefficient
LOMO: Leave-one-molecule-out
