# Supplementary figures and images for: HLA-Clus: HLA class I clustering based on 3D structure
Source: BMC Bioinformatics. 2023 May 9;24:189. doi: 10.1186/s12859-023-05297-x (PMC10169335; doi:10.1186/s12859-023-05297-x)

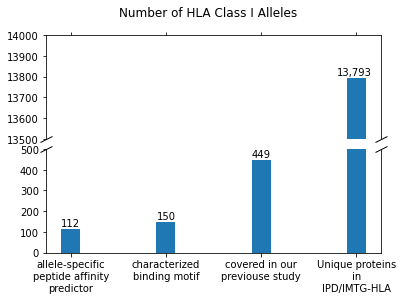

Supplement: Supplementary file 1 — Additional file1. Figure S1: Comparison between the number of HLA class I alleles studied previously. [file 12859_2023_5297_MOESM1_ESM.png]
